# Characterization of lipidome alterations in a standardized porcine model with multiple trauma and hemorrhagic shock: Are they driven by hepatic injury?

**DOI:** 10.1097/TA.0000000000004747

**Published:** 2025-08-13

**Authors:** Yannik Kalbas, Felix K.L. Klingebiel, Yohei Kumabe, Sascha Halvachizadeh, Michel P.J. Teuben, Andreas J. Hülsmeier, Christian T. Hübner, John Ricklin, Jakob Hax, Sonja Märsmann, Michal J. Okoniewski, Miriam Weisskopf, Nikola Cesarovic, Frank Hildebrand, Thorsten Hornemann, Roman Pfeifer, Paolo Cinelli, Hans-Christoph Pape

**Affiliations:** From the Department of Trauma Surgery, University Hospital Zurich (Y.K., F.K.L.K., S.H., M.P.J.T., C.T.H., J.R., S.M., R.P., P.C., H.-C.P.); Harald-Tscherne Laboratory for Orthopaedic and Trauma Research (Y.K., F.K.L.K., Y.K., S.H., M.P.J.T., C.T.H., J.R., J.H., S.M., R.P., P.C., H.-C.P.); Institute of Clinical Chemistry, University Hospital Zurich, University of Zurich (A.J.H., T.H.); Scientific IT Services ETH Zurich (M.J.O.), ETH Zurich; Center for Preclinical Development (M.W.), University Hospital of Zurich, University of Zurich; Department of Health Sciences and Technology (N.C.), Swiss Federal Institute of Technology, Zurich, Switzerland; and Department of Orthopaedic Trauma and Reconstructive Surgery (F.H.), University Hospital RWTH, Aachen, Germany.

**Keywords:** Trauma, hemorrhagic shock, liver, lipidomics, pigs

## Abstract

**BACKGROUND:**

Recent advances in analytic technology enable the investigation the response to severe injury and hemorrhagic shock (HS) can be characterized on a molecular level. While metabolomic and proteomic approaches are being actively applied in trauma research, lipidomics are less well explored.

**METHODS:**

Fifty-two male pigs were randomized to two conditions: Group PT (polytrauma) with blunt chest trauma, liver laceration, femoral fracture, and a 60-minute pressure-controlled HS and Group IF with an isolated femur fracture. Venous samples were taken from six time points (baseline, trauma, resuscitation, 2 hours, 4 hours, and 6 hours). Lipidomic analyses were performed using liquid chromatography coupled mass spectrometry. Findings were collated with standard clinical markers and near-infrared spectrometry measurements for organ perfusion.

**RESULTS:**

We identified 303 distinct lipid species that were organized in 17 functional classes. Significant group differences in lipidome dynamics were identified for the majority of lipid classes. Most notably, lipid classes involved in lipoprotein synthesis showed significant reduction over time in Group PT only. These findings collated with a significant decrease of liver perfusion during HS and a significant (~20-fold) increase of transaminases.

**CONCLUSION:**

Polytrauma with HS induces a global decrease of the circulating lipidome through acute (ischemic and mechanical) liver injury, leading to a disruption of lipoprotein synthesis. This decrease might be of clinical significance for multiply injured patients in a posttraumatic energy-depleted state and collation with clinical data, and global metabolomic analyses should be performed.

## Abstract 2

Lipidomic analysis in a porcine polytrauma model with hemorrhagic shock reveals a global dysregulation of lipid metabolism. This is most likely due to a disruption of lipoprotein synthesis as a result of ischemic and traumatic hepatopathy

## Graphical abstract

**Figure:**
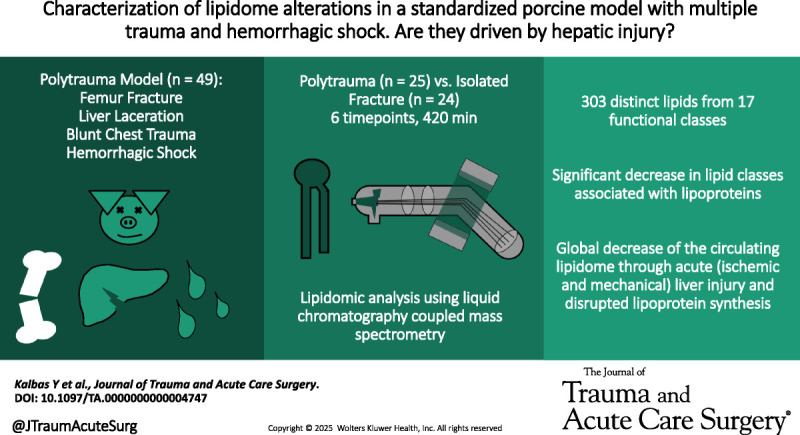

Physical trauma remains one of the leading causes of death and disability worldwide, especially in young adults.^1^ Predominantly high-energy impacts such as high falls and road traffic accidents, often cause a potentially lethal combination of severe blood loss, devastating tissue injuries, and major fractures, which require prompt surgical intervention.^2^ The systemic response to severe injury and hemorrhage has long been a major focus in trauma research, and the pathophysiological pathways are well described.^3,4^ These include the cellular and humoral inflammatory response,^5^ the pivotal role of damage associated molecular patterns,^6^ and the interplay of pathways of coagulation, acid base changes, and hypothermia.^4^

While the metabolomic reaction to trauma is currently being actively researched by several groups, the role of lipid mediators has been hypothesized.^7^ The recent advancement of analytic tools, especially mass spectrometry, allows a comprehensive characterization of circulating metabolic substrates and lipids.^8^

Lipids are a structurally and functionally diverse group, which make up the main portion of cell membranes, function as signaling molecules, and play a major role as energy substrates.^9^ Also, lipids have been shown to play an important role as regulators of (chronic) inflammatory and immune response.^10^ In addition, relevant lipid profile dynamics have been documented in patients with critical illness. Specifically, lipolysis and lipogenesis as well as circulating levels of glycerolipids, sphingolipids, phospholipids, and lysophospholipids are altered in patients with acute critical illness.^11^

Lipidomic research in trauma, however, is sparse. Analyses from a large resuscitation trial have shown that lipidome profile changes are associated with injury severity and outcome.^12^ The two main caveats in clinical studies with severely injured patients are the heterogeneity of the patient's cohort and the variability of the injury pattern. Additionally, patients are treated with a variety of therapeutic options depending on the pattern and severity of their injuries. Several standardized large animal trauma models have shown relevant changes to proteome and metabolome dynamics in response to major trauma.^13^ In a previous study, our group has shown that multiple injuries and hemorrhagic shock (HS) are associated with a systemic release of mitochondrial fatty acid metabolites in a large animal model.^14^ In this current study, our group aims to characterize the entire lipidome and its dynamics in response to polytrauma, resuscitation, and operative stabilization. We have used lipidomic analyses on plasma samples from an experimental large animal polytrauma model with blunt chest trauma, liver laceration, femoral fracture, and a mean arterial pressure (MAP)–controlled HS. We hypothesize that polytrauma with HS induces distinct lipidome profile changes and aim at investigating the following questions:

1. What is the influence of HS on the lipid metabolism?
2. Can we identify specific lipidome dynamics in response to severe injury and HS?
3. Are these dynamics in line with the systemic posttraumatic response?

## MATERIALS AND METHODS

The reporting of this study is in strict accordance with the animal research: reporting of in vivo experiments 2.0 guidelines.^15^ Lipidomic analyses were performed on plasma samples from a well-established translational porcine polytrauma model with HS. Experimental protocols were approved by the local veterinary office (license number ZH 138/2017), and the experiments were executed in accordance with federal Animal Protection Law and the “The Principles of Laboratory Animal Care.” The analyses in the current work were performed secondarily, and several previous publications already describe the methods of this porcine polytrauma model in great detail.^14,16,17,18,19^ The following paragraphs provide a summary of the experimental workflow. A comprehensive overview of the animal model including each item of the animal research: reporting of in vivo experiments 2.0 guidelines for the reporting of animal research is provided in Supplemental Digital Content (Supplementary Data 2, http://links.lww.com/TA/E721).

### Animal Model

**Figure 1:**
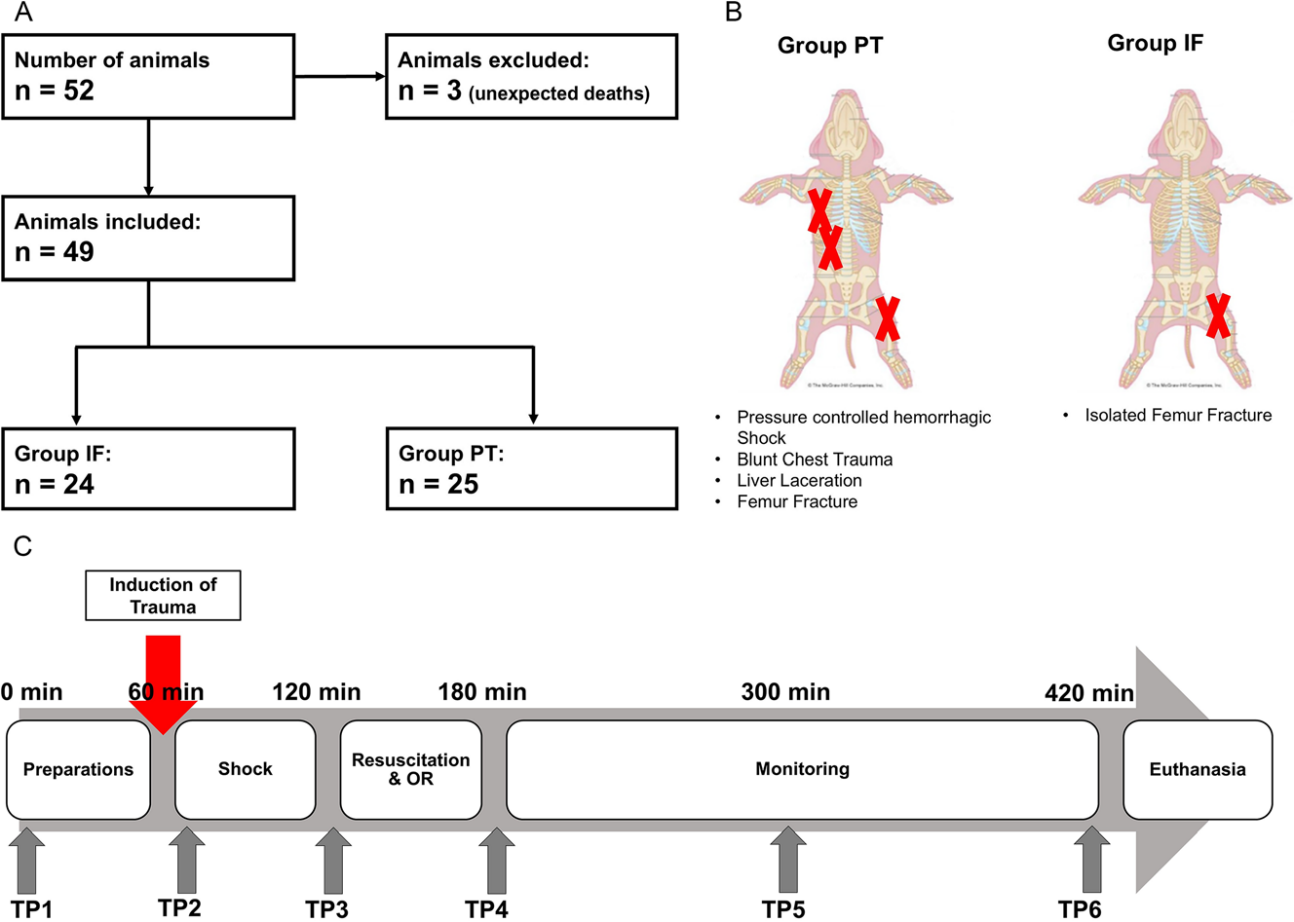
(*A*) Flowchart of animal selection process for study groups. (*B*) Schematic representation of trauma model. (*C*) Timeline.

Fifty-two castrated male Swiss large white pigs were randomized to one of the two conditions: polytrauma (Group PT) or isolated femur fracture (Group IF) as seen in Figure 1*A*. All animals were anesthetized for the duration of the experiment (induction with ketamine, midazolam, and atropine and maintenance with propofol and sufentanil forte). Group IF received an isolated femoral shaft fracture, while Group PT received an additional blunt chest trauma, a grade II (American Association for the Surgery of Trauma) liver laceration, and a pressure-controlled HS for 60 minutes (MAP, 30 mm Hg). The experimental setup is visualized in Figure 1*B*. At the end of the shock period, animals were resuscitated according to established trauma guidelines (Advanced Trauma Life Support^20^ and Arbeitsgemeinschaft der Wissenschaftlichen Medizinischen Fachgesellschaften S3 Guidelines on Treatment of Patients with Severe and Multiple Injuries^21^) by substitution of three times the withdrawn blood volume with Ringer's solution. Fractures received closed reduction and stabilization with intramedullary nailing. Observation period included 6 hours after the induction of trauma. Samples were taken from “baseline” (0 minutes), after induction of polytrauma/fracture (60 minutes), after beginning of resuscitation (120 minutes), after resuscitation and operative fracture fixation (180 minutes), and 4 hours (300 minutes) and 6 hours (420 minutes) after trauma. The experimental timeline is shown in Figure 1*C*.

### Lipidomic Analyses

Ethylenediaminetetraacetic acid-treated plasma samples were vertically placed on ice for approximately 30 minutes. Samples were then centrifuged at 1,500*g* for 12 minutes at 4°C. For lipidomic analysis, 200 μL of supernatant was aliquoted into untreated Eppendorf tubes and stored at −80°C. Lipid extraction was performed as previously described with some modifications.^22^ The MMC solvent (methanol/methyl tert-butyl ether/chloroform, 4:3:3, v:v:v) was supplemented with the SPLASH mix internal standard and additional internal standards: d7-sphinganine (SPH d18:0), d7-sphingosine (SPH d18:1), dihydroceramide (Cer d18:0/12:0), ceramide (Cer d18:1/12:0), deoxydihydroceramide (Cer m18:0/12:0), deoxyceramide (Cer m18:1/12:0), and glucosylceramides (GlcCer d18:1/18:1 (d5)) (Avanti Polar Lipids, Alabaster, AL). Lipids were extracted using a Thermomixer (Eppendorf, Hamburg, DE) at 37°C (1,400 rpm, 60 minutes) and separated using a C30 Accucore LC column (150 mm × 2.1 mm, 2.6 μm particle size) connected to a Transcend UHPLC pump (Thermo Fisher Scientific, Waltham, MA). Liquid chromatography was performed with acetonitrile/water (6:4) with 10 mM ammonium acetate and 0.1% formic acid (A) and isopropanol/acetonitrile (9:1) with 10 mM ammonium acetate and 0.1% formic acid (B) at a flow rate of 0.260 mL/min. The following gradient was applied: (*a*) 70% A/30% B, 0.0 to 0.5 minutes; (*b*) 57% A/43% B, 0.5 to 2.0 minutes; (*c*) 45% A/55% B, 2.0 to 3.30 minutes; (*d*) 25% A/75% B, 3.30 to 12.0 minutes; (*e*) 100% B, 12.0 to 25.0 minutes; and (*f*) 70% A/30% B, 25.00 to 29.50 minutes. Untargeted data acquisition was performed on a high-resolution Q-Exactive MS analyzer (Thermo Fisher Scientific, Waltham, MA). MS2 fragmentation was based on data-dependent acquisition. Lipid identification criteria were as follows: (*a*) an MS1 mass accuracy of 5 ppm from the predicted mass at a resolving power of 70,000 at 200 m/z, (*b*) isotopic distribution, (*c*) fragmentation pattern, and (*d*) expected retention time relative to internal and external standards. Data were analyzed using Tracefinder 5.1 (Thermo Fisher Scientific) for peak picking, annotation, and matching the in-house lipid database. Quality control (QC) was performed using the methods described by Broadhurst et al.^23^ with minor modifications. In brief, QC samples were prepared as pooled mixtures of every sample in five concentrations (0.05×, 0.1×, 0.25×, 0.4×, and 0.5×). Quality control samples were run as 10-fold replicates for each concentration. Two measures were used to ensure sample quality: for each lipid, the correlation coefficient between expression and sample concentration was calculated, and an *R*^2^ of >0.65 and a positive slope were defined as cutoffs. Furthermore, the coefficient of variation (%) was calculated for each QC concentration for each lipid. Lipids were only considered for further analysis if a coefficient of variation of <0.6 was found in three of the five QC concentrations.

### Measurement of Liver Tissue Perfusion

Hepatic tissue perfusion was assessed using the O2C method (Oxygen to See; LEA Medizintechnik GMBH, Giessen, Germany) as previously described.^24^ The O2C evaluates capillary-venous microcirculation with laser spectroscopy and white light spectrometry with a penetration depth of 4 to 8 mm. In Group PT, which previously underwent a laparotomy to induce the liver laceration, a probe was placed directly on the right lobe of the liver. Measurements included tissue blood flow (μFlow), oxygen saturation, hemoglobin concentration (μHb), and flow velocity. Unit of measurements is arbitrary units. Continuously, measurements were performed every second, and the average was automatically calculated after 1 minute. Liver oxygen supply was approximated, using the product of arterial oxygen saturation (SaO_2_), tissue hemoglobin concentration, tissue blood flow, and 1.34 (oxygen-binding capacity of hemoglobin) in the following formula: liver oxygen supply = SaO_2_ × μHb × μFlow × 1.34.^25^ During the induction of trauma/shock, there were very limited capacities for measurements. Therefore, the measurements of tissue perfusion were performed at 75 minutes instead of 60 minutes. Aside from this, measurements of tissue perfusion were performed at approximately at the same time as blood draws.

### Statistical Analyses

Statistical analyses were performed exclusively using R (Vienna, Australia) R-Studio (Boston, MA) as user interface.^26^ The complete code with annotations is provided in an R-Markdown script in Supplemental Digital Content (Supplementary Data 6, http://links.lww.com/TA/E725). As lipidomics data sets are typically right skewed because of occasional (very high) outliers from measurement errors, we performed outlier detection and deletion using the median *z* score method. A cutoff of −3 to 3 median *z* scores was chosen. This method was used for lipidomic data only. To account for missing values, we programmed linear mixed models for every parameter. A *p* value of 0.05 was chosen as the threshold for statistical significance. All *p* values were corrected for multiple testing using the Benjamini-Hochberg method.

## RESULTS

### Trauma Model

In total, we were able to assemble 279 samples from 49 animals. Three animals were excluded because of unexpected deaths during induction of anesthesia and/or trauma (n = 3). No significant differences between Group PT and Group IF were observed at baseline, regarding both physiological/laboratory parameters and baseline values of lipid expression as shown in Table S1 in Supplemental Digital Content (Supplementary Data 2, http://links.lww.com/TA/E721).

**Figure 2:**
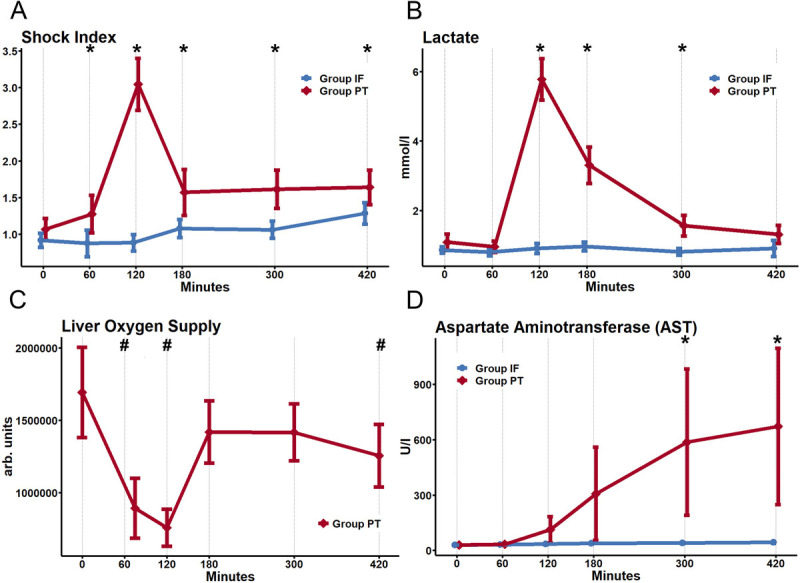
Selection of measurements to quantify the effects of HS: (*A*) Shock Index, (*B*) lactate, (*C*) liver oxygen supply, and (*D*) aspartate aminotransferase. A comprehensive overview of all relevant parameters is shown in Figure S1 in Supplemental Digital Content (Supplementary Data 2, http://links.lww.com/TA/E721).*Significant difference between groups. #Significant difference with baseline in O2C measurements.

The effects of HS are visualized in Figure 2*A* to *D* and Figure S1 in Supplemental Digital Content (Supplementary Data 2, http://links.lww.com/TA/E721; Supplementary Data 3, http://links.lww.com/TA/E722). These figures show changes over time for relevant vital parameters,^17,18^ laboratory markers,^17,18^ and measurements of tissue microcirculation. During HS induction, significant alterations of MAP, shock index, and serum lactate in Group PT could be observed (Fig. 2*A* and *B*). These values showed a relative normalization after resuscitation but still remained significantly altered compared with Group IF over the entire observation period. We also noted a significant decrease of hemoglobin after resuscitation in Group PT. No significant differences in the mean arterial oxygen saturation or core body temperature were detected between the groups (panels 5 and 6, Supplementary Fig. S1, http://links.lww.com/TA/E721). Furthermore, HS induced a significant increase of blood glucose levels (panel 7, Supplementary Fig. S1, http://links.lww.com/TA/E721) indicating a short-term “metabolic” response in Group PT. Parameters specific for muscle and liver damage (Fig. 2*D*) were also significantly altered indicating a significant disruption of hepatic tissue perfusion during and after HS in Group PT.

### Lipidomic Analyses

**Figure 3:**
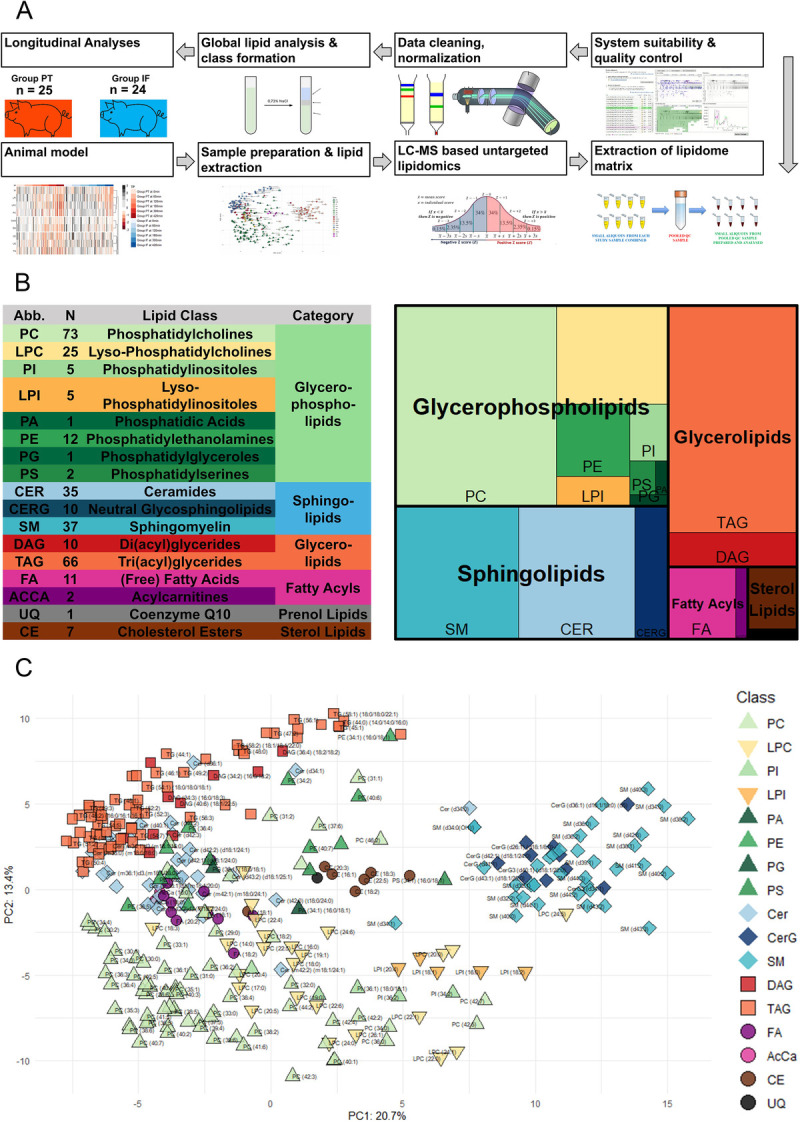
(*A*) Flowchart of the lipidomic analysis process. (*B*) Lipid ontology visualized by treemap. (*C*) PCA visualizing the variance of all lipid species for every sample. Colors and shapes represent lipid classes and categories.

We were able to generate a longitudinal data set with readouts for 565 individual lipid species. After QC, a total of 303 lipid species remained for final analyses. A flowchart of the entire workflow is presented in Figure 3*A*. According to the classification system in the LIPID MAPS Structure Database, lipid species were stratified into of six “lipid categories” (fatty acyls, glycerolipids, glycerophospholipids, sphingolipids, sterol lipids, and prenol lipids) and into 18 subclassifications, which are referred to as “lipid classes.” An overview of the lipid ontology, including the distribution of lipid species among the classes, is represented by the treemap and in the table in Figure 3*B*.

Figure 3*C* shows a principal component analysis of expression levels of individual lipid species throughout the entire data set (every sample at every time point from all animals). Colors and shapes represent lipid categories and lipid classes. Expression levels are overall very similar across lipid species from the same classes, especially classes with a smaller number of individual lipids. Larger classes show markedly more variation; however, they still form distinguishable clusters. While there are some overlaps, these commonly occur between lipid classes that are substrates of each other such as PC and LPC. Lipid categories on the other hand do not show such a level of homogeneity, which becomes especially apparent when examining the category of sphingolipids, in which Cer show a distinctive cluster, which is clearly separated from SM and CerG. In view of these findings, we chose to perform further statistical analysis based on the pooled expression of lipid species within their respective lipid class, allowing a more comprehensive overview of the relevant processes.

### Global Lipidome Analysis

**Figure 4:**
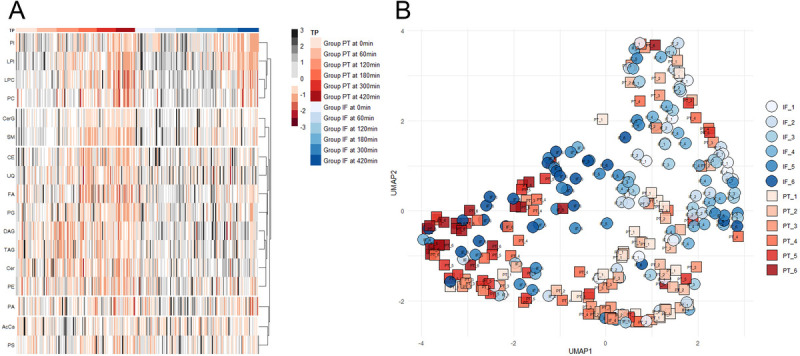
(*A*) Heatmap of all lipid classes for every time point for both groups. Rows are clustered hierarchically. Z scores calculated among all samples for each class. (*B*) Uniform manifold approximation and projection of all samples colored by time point and group.

We applied two means of dimensionality reduction to explore the alikeness and/or variance of our samples, based on study group and time point. Figure 4*A* visualizes lipid expression arranged by time point in Group PT and Group IF, respectively. Lipid classes are sorted by hierarchical clustering based on their overall similarity. *Z* scores for each lipid class in each sample are represented by the coloration. Based on this, we can already approximate lipid dynamics for clustered lipid classes. While several lipid classes (PI, LPI, PC, and LPC) show a decrease in expression over time in both groups, which is more pronounced in Group PT, many other lipid classes (CE, UQ, FA, DAG, TAG, Cer, PE, and PG) seem to decrease over time only in Group PT. Figure 4*B* depicts a uniform manifold approximation and projection of every sample, colored by time point and group. Here, we can identify a clear gradient between samples from early and late time points and a moderate distinction between the groups. It is evident that both injury severity and the time point of measurement have a relevant influence on lipid expression.

### Lipid Class Dynamics

**Figure 5:**
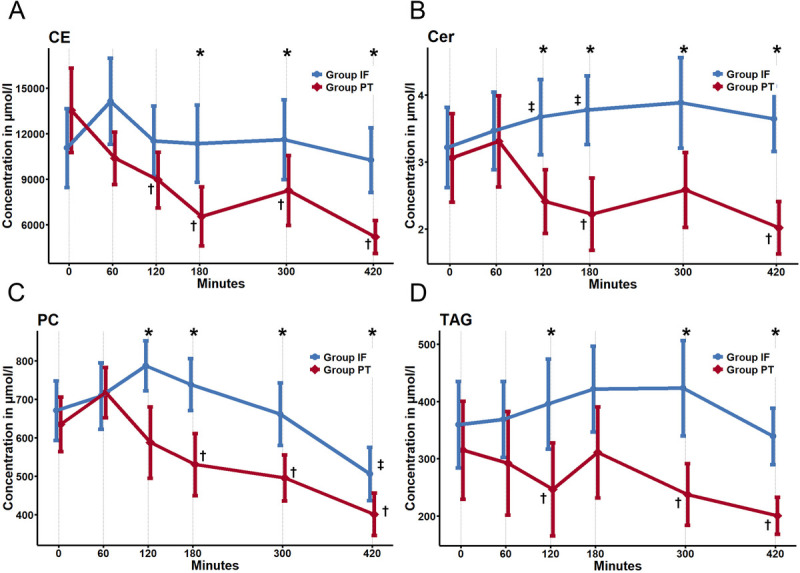
Linecharts of concentration (μM) for four select representative lipid classes: (*A*) CE, cholesterol esters; (*B*) Cer, ceramides; (*C*) PC, phosphatidylcholines; and (*D*) TAG, triacylglycerides; a comprehensive overview of all lipid classes is shown in Figure S2 in Supplemental Digital Content (Supplementary Data 2, http://links.lww.com/TA/E721). *Significant difference between groups. †Significant difference with baseline for Group PT. ‡Significant difference with baseline for Group IF.

Figure 5 and Figure S2 in Supplemental Digital Content (Supplementary Data 2, http://links.lww.com/TA/E721; Supplementary Data 4, http://links.lww.com/TA/E723) visualize longitudinal analyses for each lipid class. For many lipid classes, we could detect significant decreases in concentration over time in Group PT, while Group IF shows either a less pronounced decrease, no changes over time, or, in some classes, even an increase over time.

For CE, Cer, DAG, TAG, and UQ, we noted a significant decrease in the concentration over time for Group PT only. SM showed a significant decrease over time in both groups, which was notably more pronounced in Group PT. AcCa and PS showed a significant increase during HS in Group PT only. For most phospholipids (PA, PC, LPC, PI, LPI, and PE), we noted an initial increase in Group IF after trauma (significant for PA and PE), which was followed by a decrease throughout the observation period. For Group PT, this decrease over time was significantly more pronounced. An overview of the dynamics for each lipid class is provided in Table 1. Overall, we noted significant differences between groups in almost every lipid class for at least one time point. A summary of all statistical findings is provided in Supplemental Digital Content (Supplementary Data 2, http://links.lww.com/TA/E721; Supplementary Data 5, http://links.lww.com/TA/E724).

**TABLE 1:** Summary of Lipid Class Dynamics

Lipid Class	0 min	60 min	120 min	180 min	300 min	420 min	Summary	Component of LDL/VLDL
TAG	- / - / -	- / - / -	⇊ / - / ⇊	- / - / -	⇊ / - / ⇊	⇊ / - / ⇊	Decrease in Group PT only	Yes
CE	- / - / -	- / - / -	- / - / ⇊	⇊ / - / ⇊	⇊ / - / ⇊	⇊ / - / ⇊	Decrease in Group PT only	Yes
DAG	- / - / -	⇊ / - / -	⇊ / - / ⇊	⇊ / ⇈ / -	⇊ / - / -	⇊ / - / ⇊	Decrease in Group PT only	Yes
Cer	- / - / -	- / - / -	⇊ / ⇈ / -	⇊ / ⇈ / ⇊	⇊ / - / -	⇊ / - / ⇊	Decrease in Group PT only	Bound to LDL/VLDL
UQ	- / - / -	- / - / -	- / - / -	⇊ / - / ⇊	- / - / -	⇊ / - / ⇊	Decrease in Group PT only	Not reported
PC	- / - / -	- / - / -	⇊ / - / -	⇊ / - / ⇊	⇊ / - / ⇊	⇊ / ⇊ / ⇊	Decrease in Group PT markedly more pronounced	Yes (but esp. HDL)
LPC	- / - / -	- / - / -	⇊ / - / ⇊	⇊ / - / ⇊	- / - / ⇊	⇊ / ⇊ / ⇊	Decrease in Group PT markedly more pronounced	Small percentage
SM	- / - / -	- / - / -	- / - / ⇊	⇊ / ⇊ / ⇊	- / ⇊ / ⇊	⇊ / ⇊ / ⇊	Decrease in Group PT markedly more pronounced	Not reported
PI	- / - / -	- / - / -	- / - / -	- / - / ⇊	- / ⇊ / ⇊	- / ⇊ / ⇊	Decrease in Group PT more pronounced	Not reported
LPI	- / - / -	- / - / -	⇊ / - / ⇊	- / - / ⇊	- / ⇊ / ⇊	- / ⇊ / ⇊	Decrease in Group PT more pronounced	Not reported
PA	- / - / -	- / ⇈ / -	- / ⇈ / -	⇊ / - / -	- / - / -	- / - / ⇊	Decrease in Group PT more pronounced	Not reported
PE	- / - / -	- / ⇈ / -	- / ⇈ / -	⇊ / - / -	- / - / -	- / - / ⇊	Decrease in Group PT more pronounced	Not reported
PG	- / - / -	- / - / -	⇊ / - / ⇊	- / - / -	- / - / ⇊	⇊ / ⇊ / ⇊	Mixed dynamics	Not reported
CerG	- / - / -	- / - / -	- / - / -	⇊ / - / ⇊	- / - / -	⇊ / - / ⇊	Mixed dynamics	Not reported
FA	- / - / -	- / - / -	- / ⇈ / -	⇊ / - / ⇊	- / - / -	⇊ / - / ⇊	Mixed dynamics	Bound to albumin
AcCa	- / - / -	- / - / -	⇈ / - / ⇈	- / - / -	- / ⇊ / -	- / - / -	Increase in Group PT after trauma and HS	No
PS	- / - / -	- / - / -	- / - / ⇈	- / - / -	- / - / -	- / - / -	Increase in Group PT after trauma and HS	Not reported

Presented for each time point as 1/2/3: 1, significant difference between groups; 2, significant dynamic in Group IF compared with baseline; 3, significant dynamic in Group PT compared with baseline.

- indicates not significant.

## DISCUSSION

Polytrauma and HS are associated with a plethora of systemic pathophysiologic reactions that influence blood homeostasis, inflammatory response, and end-organ function. New analytical tools have enabled the investigation of the metabolic response to polytrauma and HS on a molecular level.^13^ The role of the lipidome in the posttraumatic reaction, however, has not yet been investigated in detail.

In this study, we created a comprehensive longitudinal lipidomic data set and identified multiple relevant alterations to the circulating lipidome. Our main findings are as follows:

- Polytrauma and HS are associated with a substantial increase of anaerobic metabolism, a significant decrease in liver tissue perfusion, and a considerable and significant increase in circulating aspartate aminotransferase (AST).
- Polytrauma and HS are associated with systemic, dynamic alterations to the circulating lipidome, which apply to the majority of lipid classes.
- For many lipid classes, a significant decrease over time occurred in Group PT only.
- Lipid classes, which decrease only in Group PT (CE, Cer, DAG, TAG, and PC) occur in circulation mainly as components of lipoproteins.

Our first finding is in accordance with various important publications in the field and with the pathophysiology of HS.^3,4^ In our study, Group PT shows a highly significant increase of serum lactate during HS, which trends to normalize after resuscitation. Lactate is commonly used as a marker for anaerobic metabolism, which, in the setting of trauma and HS, is likely caused by tissue ischemia.^27^ During HS, the lack of oxygen inhibits the terminal oxidation of the electron transport chain inside the mitochondria, causing a backflow of Krebs-cycle intermediates and thereby inhibiting glucose, amino acid, and lipid metabolism.^28^ To uphold the production of adenosine triphosphate, affected cells can switch to the anaerobic conversion of pyruvate to lactate. This process, however, being highly inefficient, can lead to a state of energy depletion, which is illustrated by the rapid depletion of blood glucose levels during resuscitation (panel 7, Supplementary Fig. S1, http://links.lww.com/TA/E721). It is important to note that, once oxidative equilibrium has been restored, lactate may be converted back to pyruvate; however, this part of the Cori cycle takes place predominantly in the liver.^29^

Regarding the significant disruption of liver tissue perfusion during HS, this is likely due to the severity of centralization and the significant reduction in blood pressure. While the tissue perfusion (flow) shows a relative normalization after resuscitation, we still noted a significant decrease of liver tissue oxygen saturation, which points to an ongoing ischemia (panel 9, Supplementary Fig. S1, http://links.lww.com/TA/E721). In this regard, the marked increase of AST is likely due to acute (hypoxic) liver injury, which is likely aggravated by the physical injury (grade II liver laceration).

In summary, our first main finding suggests a disruption in oxidative metabolism, which is aggravated by hypoxic and mechanical liver injury. This disruption is inferred from surrogate markers (lactate and AST); however, it has not been confirmed by additional metabolomic profiling and therefore should be considered a conjecture based on the known physiological responses to hypoxia and liver injury.

Our second major finding is a significant alteration to the systemic lipidome during and after polytrauma with HS. This was expected and coincides with current literature of metabolomic and lipidomic research.^12,13^ Several studies on various pathologies suggested that critical illness is associated with extensive alterations to the metabolic state and therefore the circulating lipidome; in this regard, acute hypoxic hepatopathy has been hypothesized as a main driver.^30^

Our third main result shows that the concentration of most lipid classes decrease significantly over time in Group PT, even though lipid concentrations were corrected for dilution effects, to counteract the copious resuscitation with crystalloids. This result matches well with the available scientific literature, as reduced concentration of many lipid classes is associated with higher injury severity and/or worse outcome.^12^ Wu et al.^12^ describe a “dramatic drop in all classes of lipids in the hyperacute phase after severe injury that was most extreme in patients destined to die” and point out that patients who went on to experience persistent critical illness showed a delayed rise in circulating DAG, TAG, and PE species.

Our fourth main finding shows that those lipid classes, which are significantly reduced in Group PT, appear to represent key components of lipoproteins (specifically low-density lipoprotein [LDL] and very low–density lipoprotein [VLDL]).^31,32,33^ The observed reduction of these lipid species in Group PT only may indicate impaired lipoprotein synthesis, potentially because of hepatic injury. Notably, the combination of these results with the previously described hepatic hypoperfusion, the II° laceration, and the (almost 20-fold) increase of circulating AST point to an acute (ischemic and mechanical) liver injury, which further emphasizes this hypothesis.^30^ While this interpretation is consistent with known physiology, the lack of accompanying metabolomic data limits confirmation of broader metabolic dysfunction. In this regard, it is important to consider the role of Cer, which, while not typically a predominant component of lipoproteins, is predominantly bound to LDL and VLDL when in circulation.^34^ Accordingly, the synthesis of LDL and VLDL is likely most affected by trauma and HS.

Considering that the roles of these lipoproteins are the allocation of CEs and TAGs to the peripheral tissues, a disruption of their synthesis further aggravates the aforementioned state of trauma-induced energy depletion, as TAGs are the main source of fatty acids to be used in β oxidation.^35^

Aside from this plausible hypothesis, however, it remains challenging to assign each lipid class dynamic in a specific cause and effect relationship. This is in part due to the multitude of their potential functions, such as the formation of lipid bilayers, energy storage, and cell-to-cell interactions, but also due to their constant restructuring, degradation, and conversion as part of the global lipid metabolism.^35^

Considering those lipid species, which decrease in Group PT and Group IF alike, it is likely that they are consumed as part of the trauma-induced metabolic response, which is triggered by the stress-related release of epinephrine and cortisol.^36^ One can expect that this response is more pronounced the higher the injury severity, explaining the steeper decrease in Group PT. A posttraumatic hypermetabolic response has been hypothesized as early as 1942, by Cuthbertson et al.,^37^ who referred to it as the “Ebb and Flow” hypothesis. The “ebb” phase was characterized by an initial hypometabolic state, which would soon be replaced by the “flow” state, in which the metabolism is vastly increased.^37^ While this hypothesis is still widely accepted, the role of the metabolic response to trauma has been underrepresented in the scientific literature. When applying this hypothesis to our results, it seems that the ebb phase is characterized by the initial lack of metabolic substrates through the depletion of quickly accessible glucose storage and a disrupted lipoprotein synthesis. The flow phase would then be characterized by a hypercatabolic state in which the initial energy deficit can be restored. Naturally, all forms of oxidative metabolism can only resume once resuscitation is completed and oxidative equilibrium is restored.^38^

Our results suggest that additional factors, such as hepatic function for the mobilization of fatty acyls, might play an important role.^30^ These considerations are also of clinical importance, especially regarding targeted “energy sustenance” for polytrauma patients in the intensive care unit. In this regard, further studies of metabolomic and lipidomic profile dynamics in severely injured patients should be carried out.

### Strength and Limitations

One important strength is the fact that we performed a focused lipidomic analysis, with strict QC and a dedicated database that was optimized for identification of lipid species. As the lipidome is part of the overall metabolome, many previous studies had included lipidomics as part of a global metabolomic analysis. We are convinced that the approach used in this study allows a more precise depiction of lipidome dynamics. This, however, stipulates an important limitation of this study, which is that it includes lipidomic data only, without accompanying global metabolomic profiling. As such, any conclusions about broader metabolic perturbations, particularly regarding glucose or amino acid metabolism, must be considered hypothesis generating rather than definitive. While our lipidomic findings are robust and biologically plausible, they do not independently establish systemic metabolic failure. Future studies should incorporate comprehensive metabolomic analysis to validate and expand upon the mechanistic insights proposed here. Another important limitation of this study is the fact that the model was designed to compare multiple conditions (different means of reaming, isolated vs. multiple trauma) simultaneously. In that regard, the lipidomic analyses in this study were a secondary approach. While this is in accordance with the principles of replace, reduce, and refine, an isolated omics-centered approach with an optimized model and a longer observation period might have rendered even clearer results.

## CONCLUSION

Our model indicates that polytrauma with HS leads to global lipidome alterations, which differ significantly from those in isolated fractures. These findings are in line with results from a clinical cohort and concur with the known metabolomic sequelae of HS.^12^ An in-depth analysis of specific lipid classes suggests an impaired lipoprotein synthesis, likely caused by acute (ischemic and mechanical) liver injury. This supports the idea of the liver as a primary driver of acute posttraumatic metabolomic derangement and might be relevant for management in the intensive care unit.^30^ Collation with clinical data and global metabolomic analyses are essential, and further analysis should be performed to investigate the role of specific lipid species and to identify potential targets for point-of-care resuscitation.
